# Delayed Tamponade After Transseptal Puncture: Focus on the Mechanism of a Rare But Life-Threatening Complication

**DOI:** 10.1016/j.jaccas.2025.103379

**Published:** 2025-04-02

**Authors:** Amitoj Singh, Gautam Gadey, Sherif B. Labib

**Affiliations:** Lahey Hospital & Medical Center, Burlington, Massachusetts, USA

**Keywords:** mitral valve, pericardial effusion, valve repair

## Abstract

Transseptal puncture (TSP) has evolved into a key step during various percutaneous interventions. We present a case of delayed TSP-related tamponade, which underscores the importance of vigilance even during commonly performed procedures. TSP is safest when the puncture is through the fossa ovalis, with increased risk in patients with certain anatomic features (eg, small or diminutive fossa). Despite standard precautions, this TSP resulted in delayed cardiac tamponade >2 hours postpuncture. An understanding of the embryologic anatomy of the interatrial septum and imaging is crucial for minimizing complications and ensuring optimal outcomes.

## Graphical Abstract

**Figure:**
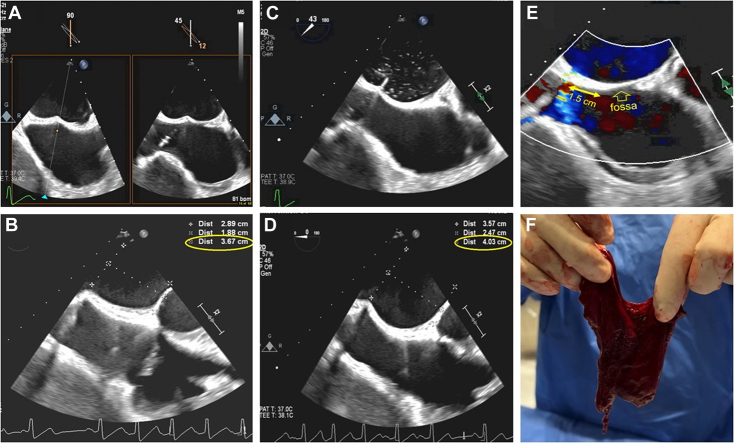

## Case Summary

**Figure 1:**
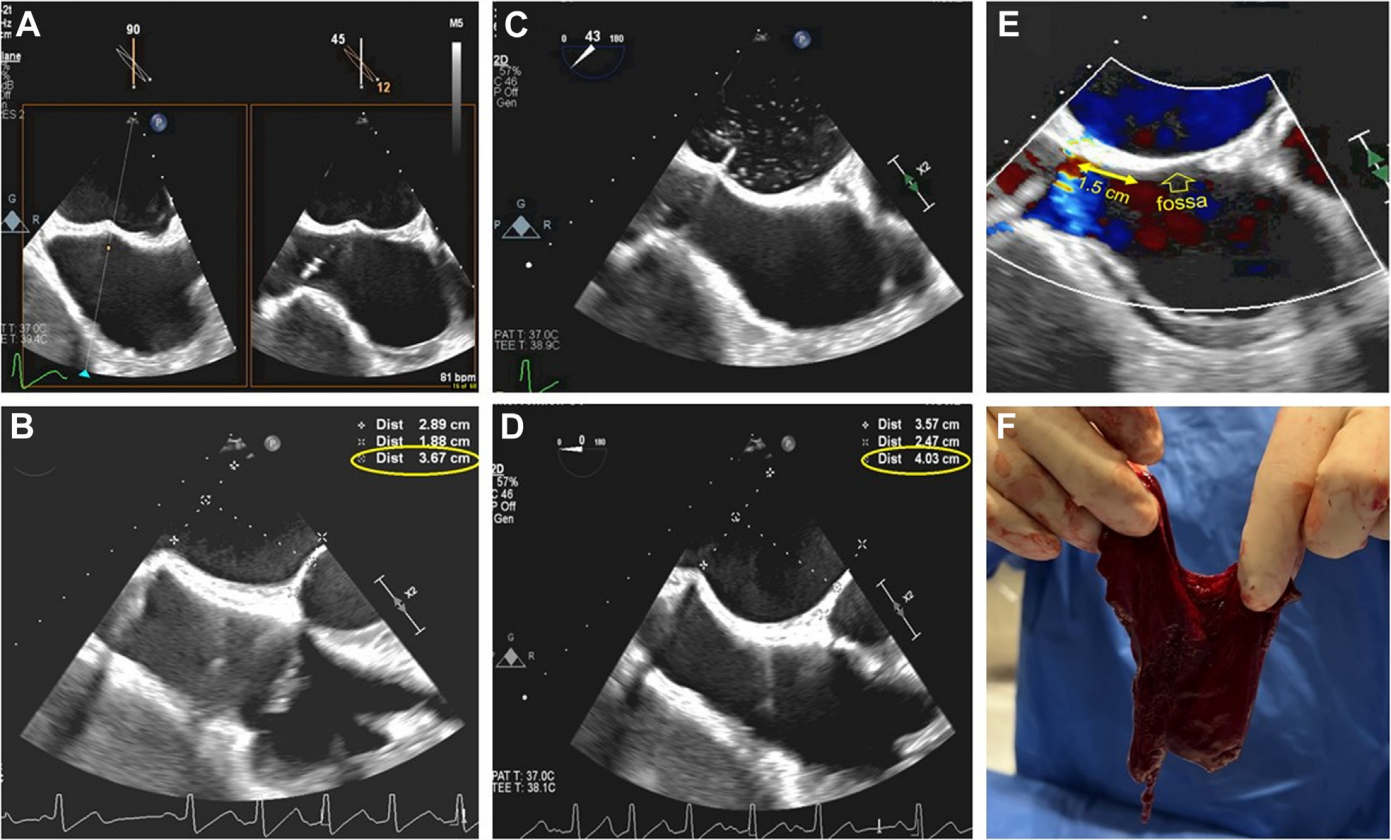
Selection of an Appropriate Transseptal Puncture Site for TEER (A) Initial tenting as viewed through X-plane imaging including short-axis orientation (at 45°). The tenting is slightly posterior to the fossa, which yielded suboptimal height for transcatheter edge-to-edge repair (TEER). (B) The distance between the initial tenting (A) and the mitral annulus (0°) is 3.6 cm, which is considered suboptimal for TEER, prompting further posterior repositing of the catheter. (C) Actual septal puncture using Baylis catheter as viewed from the short-axis orientation (43°). The trajectory and position appeared consistent with the goal of safely gaining direct access into the left atrium. The catheter tip is in a seemingly reassuring position in the left atrium. (D) The distance between the tenting and the mitral annulus (0°) is 4.0 cm after repositioning posteriorly, which is considered more appropriate for TEER, and thus selected for actual TSP. (E) Short-axis view (44°) that shows the left to right interatrial flow immediately after catheter removal. It marks the puncture site, which is in the thick portion of the septum, approximately 1.5 cm posterior to the edge of the fossa. (F) Organized thrombus extracted during emergent surgical pericardial window.

**Supplemental Figure 1:**
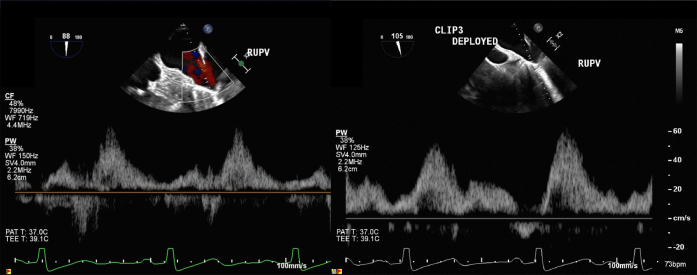
Right upper pulmonary vein flow with systolic reversal preprocedure (left panel), and fully normal Doppler wave profile with systolic predominance post MitraClip (right panel)

A 75-year-old man with symptomatic mitral regurgitation (MR) underwent transcatheter edge-to-edge repair (TEER). His history included liver and kidney transplants, and atrial fibrillation, not anticoagulated due to high bleeding risk. Transesophageal echocardiogram (TEE) revealed severe eccentric posteriorly directed MR and a large flail anterior leaflet segment. Heparin was administered before transseptal puncture (TSP). An 8.5-F VersaCross sheath (Boston Scientific Pty Ltd) was placed via the right femoral vein. A Baylis radiofrequency needle was used for TSP. TEE revealed a diminutive fossa ovalis (FO) (Video 1, Figure 1). Initial tenting in the fossa demonstrated inadequate height to the mitral valve (Video 2), prompting deliberate repositioning of the puncture posterior to the fossa to gain height. Successful TSP was confirmed with imaging without immediate complications (Video 3). Three MitraClips were ultimately deployed reducing MR from severe to mild with normalization of pulmonary vein flow pattern (Videos 4 and 5; Supplement Figure 1). Trace pericardial effusion first appeared after the second MitraClip deployment (111 minutes after TSP), which remained unchanged in size (2-4 mm) throughout deployment of a third clip (Videos 6 and 7). The effusion abruptly increased 20 minutes later as soon as the guide was pulled back into the right atrium, causing chamber compression and hypotension (Video 8). Pericardiocentesis was ineffective due to clotted blood, prompting emergent surgical pericardial window in the catheterization laboratory with full resolution of the effusion. A septal occluder was considered but deferred due to bleeding cessation and uncertain efficacy, although theoretically appealing. The patient was discharged in stable condition 6 days after the procedure.

**Take-Home Messages**

• This complication highlights the risk of entering the left atrium outside the FO, which in this case was diminutive, and was prompted by a deliberate attempt to overcome the suboptimal height required for TEER.
• The risk of tamponade is not limited to the time of TSP, but can be delayed, and occurs at the time of catheter removal across the IAS during the final step of an interventional procedure.

## Discussion

TSP is a safe technique with broad applications in various interventional procedures. A thorough understanding of interatrial septum (IAS) anatomy is essential to minimize and manage complications. The ideal puncture site varies by procedure. For degenerative MR, at least 4 cm above the mitral annulus is recommended. TSP can cause complications, particularly in cases with unusual septal anatomy. Mortality is rare (0.15%), primarily due to cardiac tamponade.

**Figure 2:**
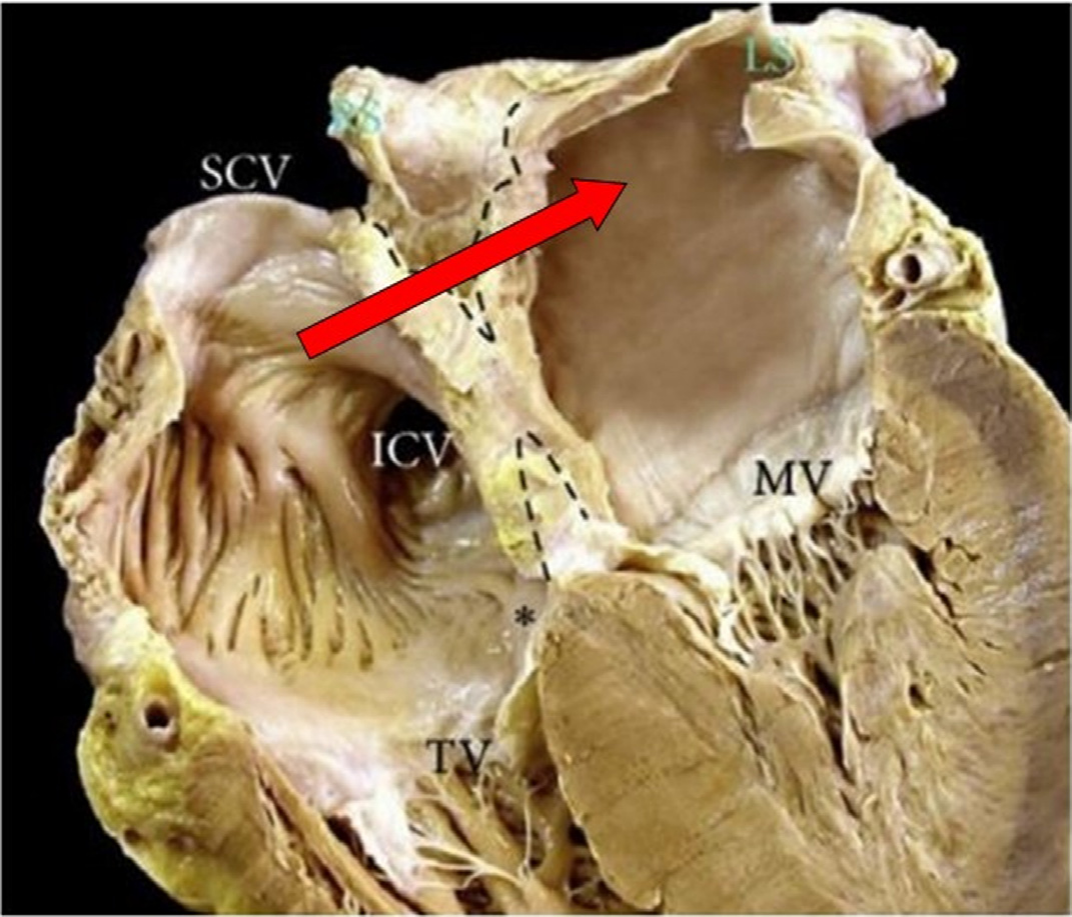
Interatrial Septum Anatomy An autopsy specimen dissected to illustrate that the thick portion of the atrial septum, which embryologically represents the septum secundum, and is also referred to as muscular septum or limbus, is not necessarily a single layer of solid tissue as it may appear on imaging. Due to the embryologic origin of the septum secundum from enfolding of atrial wall tissue on the right side of septum primum, an extracardiac space is interposed between the right and left atria walls (dotted lines). The solid red arrow depicts the potential path of a catheter if advanced from the right atrial side of the septum secundum away from the fossa ovalis (septum primum tissue). The path entails an exit to an extracardiac potential space, typically filled with adipose tissue, before entry into the left atrium, thereby increasing the risk of extracardiac bleeding complications. Original figure by Sánchez-Quintana et al^1^; adapted with permission from Pillai et al.^2^ ICV = inferior cava vein; MV = mitral valve; SCV = superior cava vein.

A TSP through the FO, which typically covers 20% of the septal area or less and is composed of septum primum tissue, provides direct interatrial communication, and should be the target for TSP. In contrast, a puncture in the septum secundum (thick portion of the IAS which forms by atrial wall enfolding during embryonic development) produces an exit from the right atrium into extracardiac fat before entering the left atrium, hence increased risk of extracardiac bleeding (Figure 2). Tamponade via this mechanism typically manifests once the guide catheter is pulled back into the right atrium, and not while the extracardiac puncture is sealed by the engaged catheter.

Although well described in recent literature,^3^ the distinction between the 2 IAS components and relevance to TSP is often underappreciated.

## Conclusions

This case emphasizes the importance of understanding the IAS developmental anatomy to reduce the risk of this rare tamponade complication.

## Funding Support and Author Disclosures

The authors have reported that they have no relationships relevant to the contents of this paper to disclose.

## Appendix

Video 1 Transesophageal Echocardiogram Showing Small, Diminutive Fossa, Which Increases Complication Risk

Video 2 Initial Attempt With Tenting Slightly at the Posterior Aspect of the Fossa That Yielded Suboptimal Height for Transcatheter Edge-To-Edge Repair (3.6 cm)

Video 3 Actual Puncture Site Used After Repositioning the Catheter Further Posteriorly From the Fossa That Yielded an Acceptable 4.0-cm Height From the Mitral Annulus Despite the reassuring appearance of the catheter tip entry into the left atrium and microbubble position confirmation, it is this step that ultimately led to delayed tamponade approximately 2 hours later due to traversing extracardiac space that is interposed between the right and left atrial walls.

Video 4 Flail A2 With Severe Eccentric Mitral Regurgitation Also recorded are the invasive measurements of a mean left atrial pressure of 35 mm Hg and a V-wave 35 to 60 mm Hg.

Video 5 Reduction in Mitral Regurgitation From Severe to Mild

Video 6 Small Effusion After Deployment of the Third and Final Clip as the Delivery Sheath Is Being Withdrawn Across the Septal Puncture Site Back Into the Right Atrium, Which Coincided With the Onset of Abrupt Pericardial Bleeding Left to right shunting across the iatrogenic atrial septal defect shows the puncture site position outside the fossa.

Video 7 3-Dimensional En Face View of the Right Atrial Septum Showing Puncture Site as Viewed From the Right Atrium (in a Generally Acceptable Area)

Video 8 Progressive Increase in Pericardial Effusion Once the Guide Was Pulled Back Across the Puncture Site, Leading to Right Ventricular Diastolic Collapse.

## References

[1] Sánchez-Quintana D., López-Mínguez J.R., Macías Y., Cabrera J.A., Saremi F. (2014). Corrigendum to “Left atrial anatomy relevant to catheter ablation.”. Cardiol Res Pract. 2020.

[2] Pillai A., Padala S., Ellenbogen K. (2021). An unusual complication of transseptal puncture. JACC Case Rep.

[3] Faletra F.F., Leo L.A., Paiocchi V.L. (2019). Revisiting anatomy of the interatrial septum and its adjoining atrioventricular junction using noninvasive imaging techniques. J Am Soc Echocardiogr.

